# The effect of an enhanced fetal growth ultrasound protocol on pregnancy outcomes: A retrospective service evaluation within a single UK National Health Service centre between 2014 and 2022

**DOI:** 10.1177/1742271X241287925

**Published:** 2024-11-08

**Authors:** Eleanor Butterfield, Emily Skelton

**Affiliations:** 1 Frimley Health NHS Foundation Trust, Frimley, UK; 2 School of Health and Psychological Sciences, City, University of London, London, UK

**Keywords:** Fetal growth, growth assessment protocol, small-for-gestational-age, stillbirth, ultrasound

## Abstract

**Aim:**

Growth Assessment Protocol is a fetal growth initiative designed to improve antenatal detection of babies who are small-for-gestational-age and reduce stillbirths. However, its direct impact on pregnancy outcome and stillbirth rates is questioned. This service evaluation aimed to assess Growth Assessment Protocol’s influence on pregnancy outcomes at a National Health Service hospital.

**Method:**

Anonymous, maternity and ultrasound data, routinely acquired between 2014 and 2022 were extracted from clinical databases (Viewpoint, Euroking). Trends in maternity data and ultrasound scan volume were explored with descriptive statistics. Variables of stillbirth, antenatal small-for-gestational-age detection and scan volume were compared before and after Growth Assessment Protocol implementation. Associations between these variables were evaluated using Spearman’s rho.

**Results:**

The percentage of babies born small-for-gestational-age reduced by 0.3% across the evaluation period. Antenatal small-for-gestational-age diagnosis rose from 4.1% to 14.3%. However, the number of false-positive cases of antenatally diagnosed small-for-gestational-age increased fivefold from 2.2% to 11.5%. Although stillbirth rates remained consistent post-Growth Assessment Protocol, complex scan volume (e.g. number of growth scans using Doppler) increased annually. The peak incline coincided with the Growth Assessment Protocol implementation period (2016–2018). Complex scan volume was significantly associated with overall small-for-gestational-age detection (rho = 0.8, *p* =< 0.001), but not with stillbirth frequency (rho = −0.1, *p* = 0.4).

**Conclusion:**

Small-for-gestational-age detection increased following Growth Assessment Protocol implementation, although this was associated with a high false-positive rate and no reduction in stillbirths. The potential implications associated with clinical management, parent experiences and departmental workflow, alongside the benefits for stillbirth reduction, should be fully considered prior to the introduction of a new fetal growth initiative to the antenatal care pathway.

## Introduction

In the United Kingdom, stillbirth is defined as the loss of a baby after 24 completed weeks of pregnancy and is predicted to occur for 1 in every 200 pregnancies.^1^ In 2016, National Health Service (NHS) England produced a national guidance called ‘Saving Babies Lives, A Care Bundle for Reducing Stillbirths’ (SBLs), to support a government pledge to halve stillbirth rates by 2030.^2^

Suboptimal fetal growth has been associated with increased risk of adverse neonatal outcome, including stillbirth.^3^ Antenatal detection of babies who are small-for-gestational age (SGA) is thought to reduce the risk of stillbirth by 50%^4^ and reduces the chance of adverse fetal outcomes when compared with babies in whom SGA has not been identified before birth.^5^

Ultrasound (US) can be used to assess fetal growth from 24 weeks gestation^6^ by calculating an estimated fetal weight (EFW) derived from fetal biometrics and plotted onto a chart for comparison against fetal growth potential for the specific gestation.^7^ It aims to identify fetuses whose EFW falls outside of the reference ranges (e.g. < 10th or > 90th percentile), particularly those who are predicted to be SGA, due to the increased risk of stillbirth within this group.

Babies classified as SGA are those born at, or predicted antenatally, to weigh <10th percentile. Babies with an EFW of <3rd percentile are defined as severe SGA.^3^ Of those, some cases will be inaccurate as they are naturally small fetuses, or the pregnancy has been incorrectly dated. However, some will present with fetal growth restriction (FGR), which occurs when there is a pathological issue, such as placental dysfunction, preventing the baby from reaching its full growth potential.^3^ Not all SGA babies will be growth restricted and vice versa; however, any baby with a predicted birthweight of <10th percentile is at a higher risk of stillbirth.^8^ A low birthweight of <2.5 kg is often associated with FGR and is therefore used as a threshold at which to determine a baby who is SGA.^3^

Traditionally, EFW is plotted on standardised growth charts (SGCs) at single timepoints and compared against a general population.^3,6^ Following the discovery of significant regional variation in UK stillbirth rates,^9^ serial growth scan protocols and more individualised care pathways were developed in the form of Growth Assessment Protocol (GAP). A key feature of GAP is the use of customised growth charts (CGCs) to estimate fetal weight. CGCs differ from SGCs, as they are devised from specific maternal characteristics such as ethnic origin and maternal size, providing an individualised chart on which to assess fetal growth at multiple time points, as opposed to SGCs which are population-based.^10^ The use of CGCs in the prediction of SGA is largely deemed positive^11^ and a paper by Hugh et al.,^4^ infers that GAP as a whole has contributed to the most significant reduction in stillbirth for the antenatal services who have implemented it. However, the true clinical value of GAP has been questioned, with published reports suggesting that antenatal detection rates of SGA babies are not improving despite the introduction of GAP^12^ and that nationally declining stillbirth rates may be coincidental to the introduction of GAP, not conclusively attributed to it.^13^

Another change to the routine growth scan as part of GAP, and subsequently an updated version of SBLs (v2) published in 2019, is the use of Doppler (an US application which uses waveform and/or colour to measure blood flow) to evaluate fetal circulation and placental function.^14^ Multiple Doppler studies, such as assessment of the umbilical and fetal middle cerebral arteries, have become integral in the assessment of fetal growth and well-being, resulting in a more complex examination.^15^ Subsequently, there has been greater reliance on growth scans to monitor fetal health, and predict fetal weight. This has resulted in increased demand for obstetric US growth scans and placed additional pressures on the sonographic workforce who required dedicated training and support to competently undertake these specialist assessments and interpret additional scan findings.^2^

A survey conducted at the National GAP user Symposium in 2019, in association with British Medical Ultrasound Society (BMUS), revealed that there is serious concern around US and Doppler capacity demands of the NHS England SBLv2 guidance, highlighting that NHS US services do not have the resources to meet the requirements.^10^

GAP was implemented at the NHS site of this service evaluation in 2016. The aim of this work was to evaluate the effect of an enhanced fetal growth US protocol (GAP) on pregnancy outcomes within this single UK NHS Centre between 2014 and 2022.

### Method

A retrospective, service evaluation enabled analysis of existing, clinical data. The Reporting of studies Conducted using Observational Routinely collected Data (RECORD) guideline was used to inform the reporting of this service evaluation^16^

Pregnancy outcome and US workflow data, routinely acquired from 2014 to 2022, were analysed. This 8-year period enabled data to be evaluated over time, starting before GAP was implemented, through implementation, covering the introduction of SBLs and beyond until 2022.

### Data collection

Data were extracted from US and maternity databases (Viewpoint and Euroking respectively) from the single UK NHS unit. The US data were retrieved using key words such as ‘growth scan’ or ‘Doppler’ along with specific dates to produce relevant data for analysis. US images were not individually reviewed for sectional data accuracy in this service evaluation; however, image auditing is routinely undertaken within the department as recommended by GAP. A data specialist acquired the maternity data on request. No prospective data were collected, and data were anonymous at the point of collection. In June 2022, the clinical site of the study undertook a hospital-wide information system transformation, resulting in the decommissioning of many of the previously used systems and databases. Therefore, data retrieved for the purpose of this study were only collected until 31 May 2022, to mitigate the potential for irregularities in the data that may be associated with the changeover and implementation of the new IT systems and databases.

Ten datasets, produced to detail per month and per year data, were collected. These included the total number of births, livebirths and stillbirths; datasets based on baby weight at birth and antenatally (<2.5 or ⩾2.5 kg); the total number of babies predicted to be SGA at birth or not (true and false positives); the number of growth scans (>24 weeks gestation) and the total number of scans which used Doppler.

Data were exported into Microsoft Excel (version 2008, Microsoft Corporation, USA) for initial curation, cleaning and analysis. Further statistical analyses were performed using IBM SPSS statistics (version 28, SPSS Inc., USA).

### Statistical analysis

Normality tests determined whether a para or non-parametric test was used. Of 10 datasets, 6 were found to be normally distributed with four not normally distributed.

Descriptive statistics for each month were calculated and reported for all datasets. This enabled 5 months in 2022 to be included in the analysis and provide further insight into scan trends and pregnancy outcome beyond 2021.

To assess for differences in pregnancy outcomes pre- and post-GAP implementation, the independent sample *t*-test or the Mann–Whitney U-test were used (subject to normality testing). All data relating to pregnancy outcome were analysed in the 2 years before GAP implementation 2014–2015 and in 2020–2021, a 2-year period after GAP implementation ensuring the intervention was completely imbedded. For correlation testing between scan volume and pregnancy outcomes, the Spearman’s rank test was used. Statistical significance was determined at the level of *p* < 0.05.

### Ethics

The NHS Research and Development team at the clinical site approved this project as a service evaluation. Approval for the project to proceed was also received by the Health Quality and Audit Team, at the clinical site. Additional approval was received by, City, University of London Research Ethics Committee (application reference ETH2223-1154, date of approval: 20.02.23).

## Results

Analyses using monthly data consisted of 101 values, representing January 2014–May 2022. Analyses using yearly data consisted of 8 values (one per year for 2014–2021), excluding the year of 2022 as it is an incomplete year of data.

### Pregnancy outcomes

In total, 45,438 births were recorded during the evaluation period. Of these, 45,288 were livebirths and 150 were stillbirths (0.3%). The total number of births per year was consistent across the evaluation period (5556 births in 2014, 5526 births in 2021). The average number of births per month was 449.9, and the average number of stillbirths per month was 1.5.

**Figure 1.**
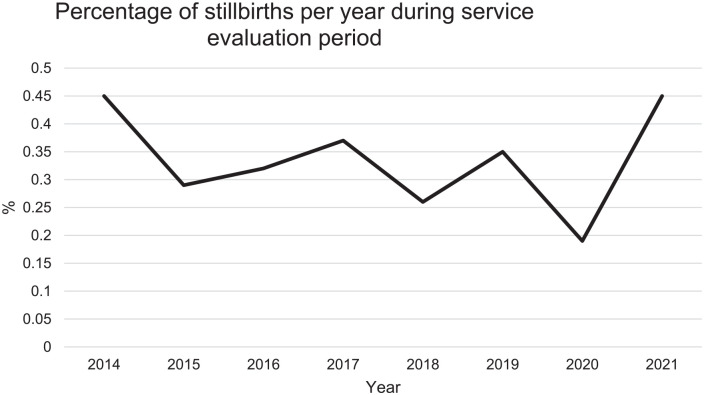
Graph demonstrating the percentage of stillbirths per year from 2014 to 2021; 2022 data were not utilised as it is incomplete.

The highest yearly stillbirth rate percentage was constant at 0.5% (*n* = 25 stillbirths) in both 2014 and 2021. However, the lowest observed stillbirth rate was 0.2% (*n* = 10 stillbirths) in 2020, with a visual decline observed prior to that (Figure 1).

### US scan volume

A total of 80,591 growth scans were undertaken during the evaluation period. Of these, 63,555 used Doppler applications. The lowest yearly scan and Doppler volumes were observed in 2014 (*n* = 5119 and *n* = 85, respectively) and the highest yearly scan and Doppler volumes were observed in 2021 (*n* = 13,409 and *n* = 13,566, respectively).

**Figure 2.**
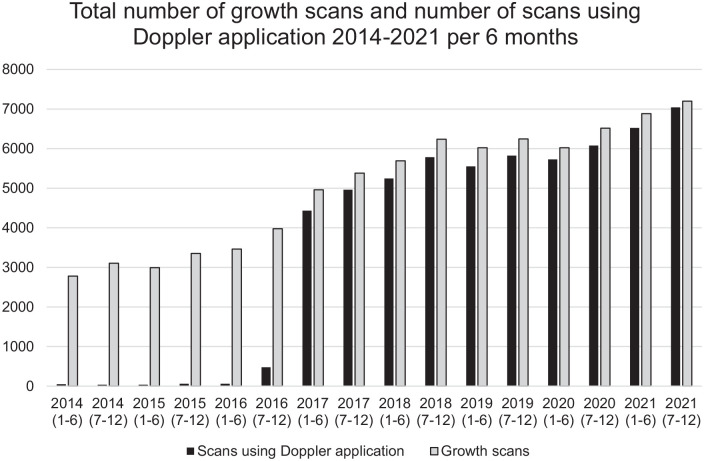
The number of growth scans and Doppler applications over the data period.

The percentage of growth scans increased annually, with nearly 50.0% of the total number of growth scans performed in the latter 3 full years of the evaluation period (i.e. from 2018). Scans during this 3-year period accounted for >60.0% of the total included in the service evaluation that used Doppler. A general upward trend in growth scan volume shows the steepest incline between 2016 and 2018, which coincides when GAP was implemented (Figure 2). It is noted that 85.9% of all growth scans recorded within the 8-year evaluation period were performed after the implementation of GAP in 2016.

### Antenatal SGA detection

On average, the number of babies born per month weighing <2.5 kg was *n* = 26 (SD = 5.83), equating to 5.8% of the total babies born each month. An average of 43.6 babies per month were antenatally predicted to weigh <2.5 kg at birth. Of those, an average of 11.6 were born weighing <2.5 kg (true-positive SGA) and 31.9 were born weighing ⩾2.5 kg (false-positive SGA). This gives a mean true-positive rate of antenatal SGA prediction of 27.0%, with a mean false-positive rate of 73.0%. Of all babies born weighing <2.5 kg, 56.2% were not detected antenatally.

**Figure 3.**
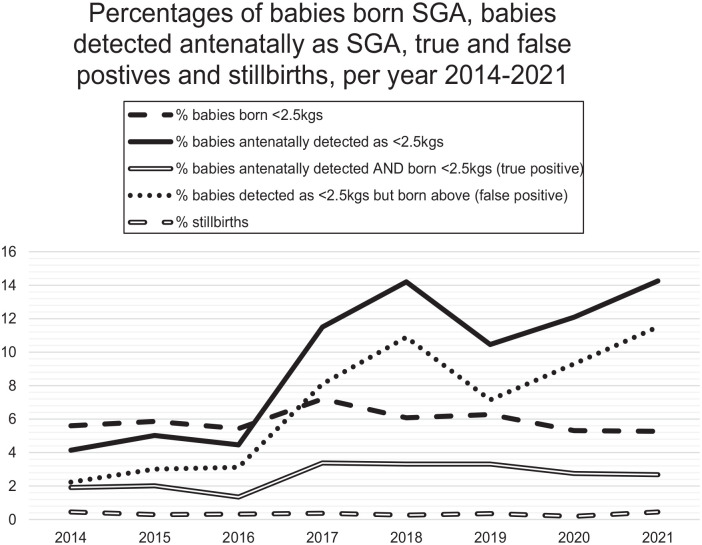
The percentages of SGA detection versus outcomes.

Figure 3 demonstrates that there was a higher percentage of babies detected as SGA in 2021(14.3%) than in 2014 (4.14%). However, there were more babies born weighing <2.5 kg in 2021 (*n* = 291) compared with those correctly detected antenatally as SGA in 2021 (*n* = 148), indicating a gap in SGA detection. An increase in false-positive detection is also demonstrated. The percentage of true-positives did increase over the evaluation period, but not at the same rate as overall antenatal SGA detection and false-positive antenatal SGA detection. Of all the babies identified as SGA antenatally in 2021 (*n* = 788), a higher number of these babies were falsely detected (*n* = 636) compared with true-positives (*n* = 148).

The percentage of babies born weighing <2.5 kg compared with the total number of livebirths reduced by 0.3% from 2014 to 2021 (5.6% to 5.3%, respectively). The percentage of babies correctly detected as SGA antenatally rose from 1.9% in 2014 to 2.7% in 2021, indicating an increase in true SGA detection by just over 40.0%. However, the proportion of babies identified antenatally as <2.5 kg but not SGA at birth (false-positives) also increased fivefold (2.2% in 2014 to 11.5% in 2021), suggesting over-detection of SGA antenatally.

### Comparison of stillbirth and SGA data pre- and post-GAP implementation

To compare data pre- and post-GAP, the two calendar years (24 months) prior to intervention and the two latest full calendar years of data available post-intervention (also 2 years), were analysed. Statistically significant differences are observed between groups. There were significantly more babies born weighing <2.5 kg in the pre-intervention period than in the post-intervention period (0.02). Conversely, there were significantly more babies detected as SGA in the post-intervention period than the pre-intervention period (*p* < 0.001), with both true (*p* = 0.02) and false-detection (*p* < 0.001) demonstrating a significant increase. The mean rank values of stillbirths demonstrated a reduction in 2020/2021 (22.4) from 2014/2015 (26.6); however, this was not statistically significant (*p* > 0.05). See Table 1 for data.

**Table 1.** Pre- and post-intervention data for stillbirths and SGA.

Variable	Epoch/pre- or post-intervention	2014–2015 (Pre)	2020–2021 (Post)
Total number of stillbirths	Mean rank	26.6	22.4
	Sum of ranks	639.0	537.0
	Z	−1.1	
	Mann–Whitney sig. *p* value (2-tailed)	0.3	
Total number of babies born weighing <2.5 kg	Mean	26.6	23.5
	Standard deviation	4.5	4.6
	*t*-test sig. *p* value (2-sided)	**0.02**	
Total number of babies antenatally detected as weighing <2.5 kg at birth	Mean	21.3	28.6
	Standard deviation	7.6	13.6
	t-test sig. *p* value (2-sided)	<**0.001**	
Total number of babies antenatally detected as weighing and born weighing <2.5 kg (true positives)	Mean rank	19.7	29.4
	Sum of ranks	471.5	704.5
	Z	−2.4	
	Mann–Whitney sig. *p* value (2-tailed)	**0.02**	
Total number of babies antenatally detected as weighing <2.5 kg but born weighing ⩾2.5 kg (false positives)	Mean	12.2	46.3
	Standard deviation	5.7	13.3
	*t*-test sig. *p* value (2-sided)	**<0.001**	

Significant values in **bold**.

### Correlations between scan volume and (a) stillbirth rates and (b) SGA detection

#### Scan volume and stillbirths

A very strong, significant positive correlation was demonstrated between scan volume and Doppler application (rho = 0.98, *p* < 0.001). Absence of other significant correlations infers that the observed increase in scan volume is not associated with the frequency of livebirths or stillbirths (Table 2).

**Table 2.** The correlation coefficient data for growth scans, Doppler application and SGA detection.

Spearman’s rank correlation coefficient	rho (*p*-value/95% CI)
Total number of babies antenatally detected as <2.5 kg and Total number of growth scans	0.8 **(<0.001**/0.7 − 0.9)
Total number of babies antenatally detected as weighing <2.5 kg and born weighing <2.5 kg (true positives) and Total number of growth scans	0.4 **(<0.001**/0.2 − 0.5)
Total number of babies antenatally detected as weighing <2.5 kg but born weighing ⩾2.5 kg (false positives) and Total number of growth scans	0.8 **(<0.001**/0.7 – 0.9)
Total number of babies antenatally detected as <2.5 kg and Total number of scans using Doppler application	0.8 **(<0.001**/0.7 − 0.9)
Total number of babies antenatally detected as weighing <2.5 kg and born weighing <2.5 kg (true positives) and Total number of scans using Doppler application	0.4 **(<0.001**/0.2 − 0.5)
Total number of babies antenatally detected as weighing <2.5 kg but born weighing ⩾2.5 kg (false positives) and Total number of scans using Doppler application	0.8 **(<0.001**/0.7 − 0.9)

CI: confidence interval.

Significant values in **bold**.

#### Scan volume and SGA detection

Higher scan volume was significantly associated with higher numbers of babies being classified as SGA (rho = 0.8 *p* < 0.001). In addition, a strong, significant correlation between complex scan volume (e.g. scan volume and Doppler application) and antenatal SGA detection was also demonstrated (rho = 0.8, *p* < 0.001) (Table 2). Of note, the strength of association between complex scan volume and false-positive detection (rho = 0.8) is greater than that of true detection, where only a moderate association is seen (rho = 0.4). This suggests that an increase in complex scans is more closely correlated with false SGA detection than true SGA detection.

## Discussion

This service evaluation found that despite the introduction of GAP in 2016, stillbirth rates at the clinical site remained unchanged between 2014 and 2022. While SGA detection increased overall, false-positive SGA detection increased far more than true-positive SGA detection. The findings also demonstrated a substantial increase in US scan volume and use of Doppler application, showing a significant, positive correlation with SGA detection, but not with stillbirth rates.

### Stillbirths

Stillbirth rates at this clinical site did not change during the evaluation period. However, a visual decline of stillbirths pre-2021 was observed in the data, despite the increase to 2014 levels in 2021. In March 2020, the COVID-19 pandemic resulted in a national lockdown, followed by a period of social restrictions in the UK for much of 2020 and 2021.^17^ Pregnant women were classed as at high-risk of severe illness from COVID-19 and were recommended to stay at home to reduce the risk of contracting the virus.^18^ Research into how COVID-19 affected pregnancy outcomes have produced differing results with Khalil et al.^19^ reporting an increase in adverse outcomes such as stillbirth rates at an NHS Trust in the South East of England. Conversely, Wilkinson et al.^20^ found no significant difference in stillbirths at a different NHS unit in the North West of England. This suggests that the effect of COVID-19 on pregnancy outcomes could be associated with other geographical factors such as staffing, localised infection severity or women’s willingness (or lack of) to access antenatal services during the pandemic. The upward trend in stillbirths seen nationally in 2021 could therefore, have been influenced by COVID-19.

Despite no observed change in stillbirth rates overall at this clinical site, there was a decline recorded by the Office for National Statistics (ONS) in the number of stillbirths recorded in England and Wales during the same data period.^21^ A similar trend was reported in Scotland.^22^ As GAP is not as widely implemented in Scotland compared with England,^23^ these results indicate that there may be other factors contributing to the decline in stillbirths. This aligns with findings of other studies, which conclude that the national stillbirth decline is likely to be related to background factors such as advances in antenatal practice or maternal health, rather than GAP.^12,13^

Outside of the United Kingdom, similar results have been found; clinical units using GAP have recorded a reduction in stillbirths which appears to coincide with a national decline. This has led to several studies concluding that background changes, such as increased worldwide awareness of factors associated with stillbirth, may have had an unknown impact on their individual results.^24–26^ In addition, a retrospective cohort study of FGR performance indicators in Australia has been associated with an increase in SGA detection and a reduction of stillbirth rates,^27^ suggesting that increased awareness, publicly and professionally, may have been a contributing factor. The potential influence of background factors reduces the ability to confidently determine what direct effect GAP has had, even if small, on the reduction in stillbirths in the United Kingdom.

### False SGA antenatal detection

An increase in false SGA detection may result in the over-diagnosis of SGA. Incorrect SGA detection has substantial implications for ongoing pregnancy management as well as potentially increasing obstetric intervention;^28^ it may contribute to an overuse of resources, particularly within US services. Further scans would usually be requested to enable continued monitoring of fetal weight,^29^ which may inadvertently exacerbate the issue of false-positive detection. In addition, increased frequency of antenatal scans and appointments have also been found to increase parental anxiety, because of worry about the baby’s growth and the associated complications of reduced growth.^29^ Indeed, Heidweiller-Schreurs et al.^30^ reported that even when parents felt the extra scans were necessary and in the best interests of the baby, they still had a significant impact on their lives, taking up more time and requiring scheduling of childcare, work commitments and extra travel, potentially adding stress.

A comparison of national guidance demonstrates differences between policies. GAP guidance^10^ recommends a minimum scan interval of 2 weeks, whereas BMUS,^31^ Royal College of Obstetricians and Gynaecologists,^3^ NHS England^14^ and International Society of Ultrasound in Obstetrics and Gynaecology,^32^ recommend that growth scans should be a minimum of 3 weeks apart, to lessen the detection of false-positive rates for fetal growth discrepancies. It could therefore be argued that the reduced scan interval of GAP may have contributed to the increase in false-positive SGA detection. In addition, different biometric reference charts have been observed to produce a range of predictive outputs using the same data, with the GAP chart GROW, predicting birthweights of <10th centile more frequently than other charts,^28^ potentially contributing to increasing false SGA detection.

Image auditing was undertaken as part of GAP, to ensure consistency and accuracy of biometry acquisition; however, vetting or the process of justifying the US requests was limited during the study period; requests were most frequently accepted without question, when requested by obstetric professionals. Vetting is commonplace for non-obstetric US scans and there is clear published guidance available; however, this is not the case for obstetric US vetting.^33^ This may have permitted clinical judgement to cause a deviation from the protocol, resulting in a less consistent growth pathway, impacting on the false positive SGA detection.

## Implications for clinical practice

The increase in complex scan volume associated with the increased SGA diagnosis, may create capacity issues for US and maternity departments. If a large proportion of fetuses are incorrectly detected as SGA, then a large proportion of associated surveillance growth scans and antenatal appointments may also be unnecessary. In addition, any increase in scanning, particularly that of multiple consecutive growth scans, places the sonographer at risk for work-related musculoskeletal disorders (WMSDs) due to repetitive movements,^34^ which can be exacerbated by the exertion required for the scanning of women with increased body mass.^35^ A sonographer with a WMSD will be unable to work, adding further pressure to the department.

The NHS is already stretched with long waiting lists and healthcare professionals have been striking throughout 2023 in a bid to reach better working conditions and increased pay, including radiographers (many of whom work in US).^36^ Within obstetrics, waiting lists cannot exist due to the time-dependent nature of examinations; therefore any requests for extra scans must be accommodated in (often) already full appointment lists. There is an acknowledgement that NHS US departments are lacking resources to accommodate requested growth scans^10^ due to a sonographer workforce vacancy rate of 12.5%.^37^ This shortage is likely to increase pressure on existing staff and may be contributing to the COVID-19 occupational burnout that has been reported at a high level in obstetric sonographers (>90% in a sample of *n* = 89), many of whom (>70%) were considering leaving the profession or changing their working practices within 5 years.^38^ A continued increase in demands for fetal growth scans is not only going to affect US capacity, but also risk exacerbating staff burnout, as workloads increase and staff shortages persist.

This increase in workload may compromise time availability to learn new skills, such as different Doppler assessments as per GAP.^10^ It is accepted that the accuracy of US is operator-dependent,^39^ therefore, it must remain of high importance that there is continuous professional development adopted by all practitioners to meet the standards required by relevant professional regulators.^40^ Regular auditing of US images should continue to be undertaken at the clinical site with transparent and constructive feedback loops, to ensure growth discrepancies are not due to operator error.^31^

Some changes, such as more robust vetting practices have now been implemented by US leadership, ensuring strict accordance with agreed protocols, helping to reduce unnecessary workload. However, these data provide an opportunity for a multidisciplinary discussion as to how clinical efficacy of US examinations can be maximised, taking into consideration all factors that influence the potential overuse of US. Improved efficiency may help to mitigate the impacts identified in this service evaluation.

## Strengths and limitations

The retrospective nature of the study data curbs the ability to generalise results. More robust results would have been yielded using data from CGCs that is, those babies plotted as EFW < 10th percentile customised for each pregnancy. However, because CGCs over the data period were paper copies, these data were unavailable for use. The study’s clinical site now uses digital CGCs on GROW software (implemented Autumn 2021), which should aid audit and analysis in future studies.

In addition, the dataset does not allow for small potential changes in fetal weight between the last recorded EFW from growth scan and the fetal weight at birth, which could result in an antenatally detected SGA recorded on 9th centile, increasing in weight such that the birth weight falls on the 10th or 11th centile. Furthermore, it was not possible in this dataset to identify individual elements which may be more effective in identifying true SGA babies. Thus, it may be beneficial for future work to include dedicated analyses of actual birth weight in comparison to the last recorded EFW and AC measurements. These could be undertaken in conjunction with image quality reviews, and consideration could also be given to the use of different growth charts or alternative biometric formulae to better understand the contribution of these factors in the classification of SGA.

Strengths of the evaluation include the use of routinely acquired data prior to the knowledge of this study. This was resourceful and advantageous to mitigate potential bias, such as confirmation and selection biases. The volume of data collected increases the statistical confidence, and the long evaluation period allows for changes to be fully observed. Robust searches of both databases ensured that all relevant data was captured.

## Conclusion

The introduction of GAP at this clinical site in 2016 changed the obstetric management pathway by introducing a more detailed and complex fetal growth surveillance protocol. In keeping with the existing literature, the findings from this service evaluation suggest that the implementation of GAP made no conclusive difference to the pregnancy outcome of stillbirth, despite an increase in true antenatal SGA detection. In addition, false-positive detection of SGA and complex scan volume were found to have increased. These findings suggest that while US and Doppler are valuable tools in the assessment of fetal growth, there are associated limitations in their use which can produce uncertainties and service issues. The findings of this service evaluation highlight the need to fully consider and balance the implications for resourcing, training, clinical management and patient experience, alongside the potential benefits for stillbirth reduction, when introducing a new fetal growth initiative to the antenatal care pathway.
